# Noninvasive Measurement of Cerebrospinal Fluid Flow in Shunted Hydrocephalus: Protocol for Scanner Calibration and Multisite Data Collection

**DOI:** 10.2196/85918

**Published:** 2026-02-20

**Authors:** Jason M Toliao, Matthew T Borzage, Pradip P Chaudhari, Joseph H Ha, Isabella Friedli, Madison E Gutierrez, Meghan Drastal, Peter Calabrese, Eamon K Doyle, Isabel Torres, Jacob Al-Husseini, Clio González-Zacarías, John C Wood, Jason Hauptman, J Gordon McComb, Stefan Blüml, Peter A Chiarelli

**Affiliations:** 1 University of Southern California, Los Angeles, CA, United States; 2 Fetal and Neonatal Institute, Division of Neonatology, Children's Hospital of Los Angeles, Los Angeles, CA, United States; 3 Alfred E Mann Department of Biomedical Engineering, Viterbi School of Engineering, University of Southern California, Los Angeles, CA, United States; 4 Department of Regulatory and Quality Sciences, Alfred E. Mann School of Pharmacy and Pharmaceutical Sciences, University of Southern California, Los Angeles, CA, United States; 5 Division of Emergency and Transport Medicine, Children's Hospital Los Angeles, Los Angeles, CA, United States; 6 Keck School of Medicine, University of Southern California, Los Angeles, CA, United States; 7 Division of Neurosurgery, Department of Surgery, Children's Hospital of Los Angeles, Los Angeles, CA, United States; 8 Stanford School of Medicine, Palo Alto, CA, United States; 9 Department of Nursing Administration, Children's Hospital Los Angeles, Los Angeles, CA, United States; 10 Department of Quantitative and Computational Biology, University of Southern California, Los Angeles, CA, United States; 11 Department of Radiology, Children's Hospital of Los Angeles, Los Angeles, CA, United States; 12 Division of Cardiology, Children's Hospital of Los Angeles, Los Angeles, CA, United States; 13 Division of Neurosurgery, Phoenix Children’s Hospital, 1919 E Thomas Rd, Phoenix, AZ, 85016, United States, 1 (602) 933-1000

**Keywords:** cerebrospinal fluid shunt, phase contrast, magnetic resonance imaging, hydrocephalus, shunt malfunction, study protocol, method, cerebrospinal fluid

## Abstract

**Background:**

Ventricular shunts divert cerebrospinal fluid (CSF) in patients with hydrocephalus, which can be lifesaving. Untreated shunt failure may lead to increased intracranial pressure and neurological injury. The process of diagnosing shunt malfunction can be complex, and historically, there has not been a simple method for noninvasive and quantitative measurement of CSF flow through shunts. The demonstration of successful clinical application of phase-contrast magnetic resonance imaging (PC-MRI) to noninvasively quantify shunt flow is relatively new and will benefit from standardization across varying types of magnetic resonance (MR) hardware to facilitate implementation at multiple medical sites. PC-MRI CSF flow measurement through ventricular shunts has not yet been compared across different types of MR hardware (ie, differing field strengths, radiofrequency coils, slew rates, gradient strengths, models, and manufacturers).

**Objective:**

This study describes a protocol aimed to standardize and optimize PC-MRI shunt flow measurements for widespread use on any MR scanner.

**Methods:**

To study shunt flow in a manner that would translate effectively to the patient care setting, a phantom model incorporating a human shunt catheter can be constructed to obtain data over a typical physiological range of CSF flow rates. Calibration curves are used to model data comparing known flow rates to flow measured via PC-MRI. The accuracy of each MR scanner is assessed using linear regression. This protocol will be repeated on 8 MR scanners before multisite data collection.

**Results:**

The shunt flow phantom was constructed in November 2024. Single-site phantom calibration began in April 2025 and concluded in December 2025. Registration of clinical data collection sites will take place between November 2025 and July 2026. Approval from the Children’s Hospital of Los Angeles Institutional Review Board was obtained in January 2023 (before scanner calibration), and single-site data collection is ongoing. Multi-institutional clinical data collection will begin in July 2026 and continue until January 2027. Results and statistical analyses are expected by April 2027.

**Conclusions:**

This study protocol provides a methodology to test and implement PC-MRI on any MR scanner using a phantom model that (1) represents real flow and catheter conditions, (2) provides a repeatable means for data collection, (3) is easy to assemble, (4) minimizes artifacts, and (5) is transportable. We also describe analytic methods for interscanner calibration across a range of hardware parameters and provide a framework for multisite data collection.

## Introduction

Over 380,000 patients worldwide are diagnosed annually with hydrocephalus [1], a pathological imbalance between cerebrospinal fluid (CSF) production and absorption [2], often causing markedly increased intracranial pressure [3,4]. If left untreated, elevated intracranial pressure can lead to severe neurological damage, disability, or death [3-5]. Placement of a ventricular shunt to treat hydrocephalus is a frequently performed neurosurgical procedure. Ventricular shunts redirect CSF to a different site in the body for absorption [3,4,6]. Mechanical obstruction or migration of shunt hardware leads to failure in nearly 30% of patients within 6 months of shunt placement [7], and more than 38,000 hospitalizations occur annually related to CSF shunt systems [8].

Evaluation of shunt function in clinical practice can include the following approaches applied independently or in combination: review of presenting signs and symptoms [3,5,9], structural imaging of ventricular size [3,10-12], x-ray imaging of hardware integrity [3,11,13,14], percutaneous shunt tap [15], radionuclide shunt flow study [16-18], or continued inpatient monitoring for evolution or resolution of symptoms [5,8]. The ability to reliably detect shunt malfunction remains imperfect. Approximately a quarter of patients with shunt failure exhibit no change in ventricular size [16], rendering accurate detection of malfunctioning shunts a composite assessment and highlighting the importance of clinical experience [5,19,20]. There is potential to improve modern clinical practice and provide additional rigor to the determination of shunt function or malfunction by measuring shunt flow noninvasively through magnetic resonance imaging (MRI) [21].

Phase-contrast magnetic resonance imaging (PC-MRI) was recently demonstrated [21-23] as a fast and reliable scan sequence to measure bulk movement of CSF in ventricular shunts. The PC-MRI technique for shunt flow measurement has been validated for multiple patients at a single institution [21], with active plans to expand use of the technology through multi-institutional implementation and data assimilation. Adoption of the scan sequence at different hospitals and on different magnetic resonance (MR) scanners requires thorough confirmation that shunt flow measurements are of comparable accuracy and reliability at these sites. Results are influenced by software (scanning features and parameters), hardware (field strength, radiofrequency coil design, gradient strength, gradient slew rate, manufacturer, and model), or both. A systematic framework to investigate these factors will assist in guiding expectations for the success of flow measurement on different scanners and will provide a potentially necessary means to account for variable bias in flow measurement if this effect were to occur on a given scanner or hardware combination. Scanner calibration and quantification of expected error will enhance the reliability of data acquired using different types of MR hardware during multi-institutional data collection and standardize PC-MRI protocols for implementation on any MR scanner.

This work presents (1) a consolidated description of the hardware variables that must be accounted for on new scanners; (2) a method for any institution to implement a simple phantom design to measure shunt flow; (3) a protocol for flow rate testing via PC-MRI on different MR scanners; (4) an appropriate suite of basic and more stringent analytic methods to calculate flow; (5) a statistical approach to determine potential hardware-induced bias, as well as to correct for bias potentially introduced on different scanners; and (6) plans to validate PC-MRI for multisite clinical data collection. By using a homogeneous phantom medium with the same hardware used in human shunts, we ensure consistent measurements that can effectively model in vivo shunt flow. The combination of a linear single-catheter phantom with a screw-driven pump mechanism allows for precise and reliable delivery of preset flows across a specific spectrum of physiological flow rates. PC-MRI flow measurements can be compared with predetermined flow rates to obtain a calibration curve for individual scanners and their particular hardware. After a thorough investigation of scanner-related variability is performed, we will collect clinical data across multiple institutions to evaluate the feasibility of implementing standardized PC-MRI data collection platforms.

## Methods

### Overview

We constructed a phantom for data acquisition and developed a standardized method to calibrate PC-MRI for accurate shunt flow assessment on any MR scanner. Our protocol applies phantom calibration to evaluate variation in PC-MRI measurements introduced by different MR hardware and validates multisite PC-MRI data collection. Rigorous methods for statistical comparison between types of scanner hardware are also provided.

### Apparatus for Data Collection

We constructed and evaluated a versatile suite of phantoms (Multimedia Appendix 1). A single-tube, linear phantom was used for data collection based on its simple design and reproducible flow production. The single-tube phantom was constructed using an antibiotic-impregnated catheter with a 1.3-mm inner diameter (Ares model; Medtronic), a common surgically placed distal shunt catheter in patients at our institution. The catheter was secured longitudinally through a rectangular 500-mL biologic media flask filled with water to eliminate air pockets and optimize the magnetic homogeneity of the imaged region.

An Alaris screw-driven syringe pump (Becton, Dickinson and Company) provided modifiable flow rates to drive water through the phantom in a continuous fashion without peristaltic or pulsatile action. The pump was connected to one end of the phantom using small-bore Luer lock tubing (ICU Medical). Materials and a step-by-step procedure for phantom construction are provided in Multimedia Appendix 1.

**Figure 1.**
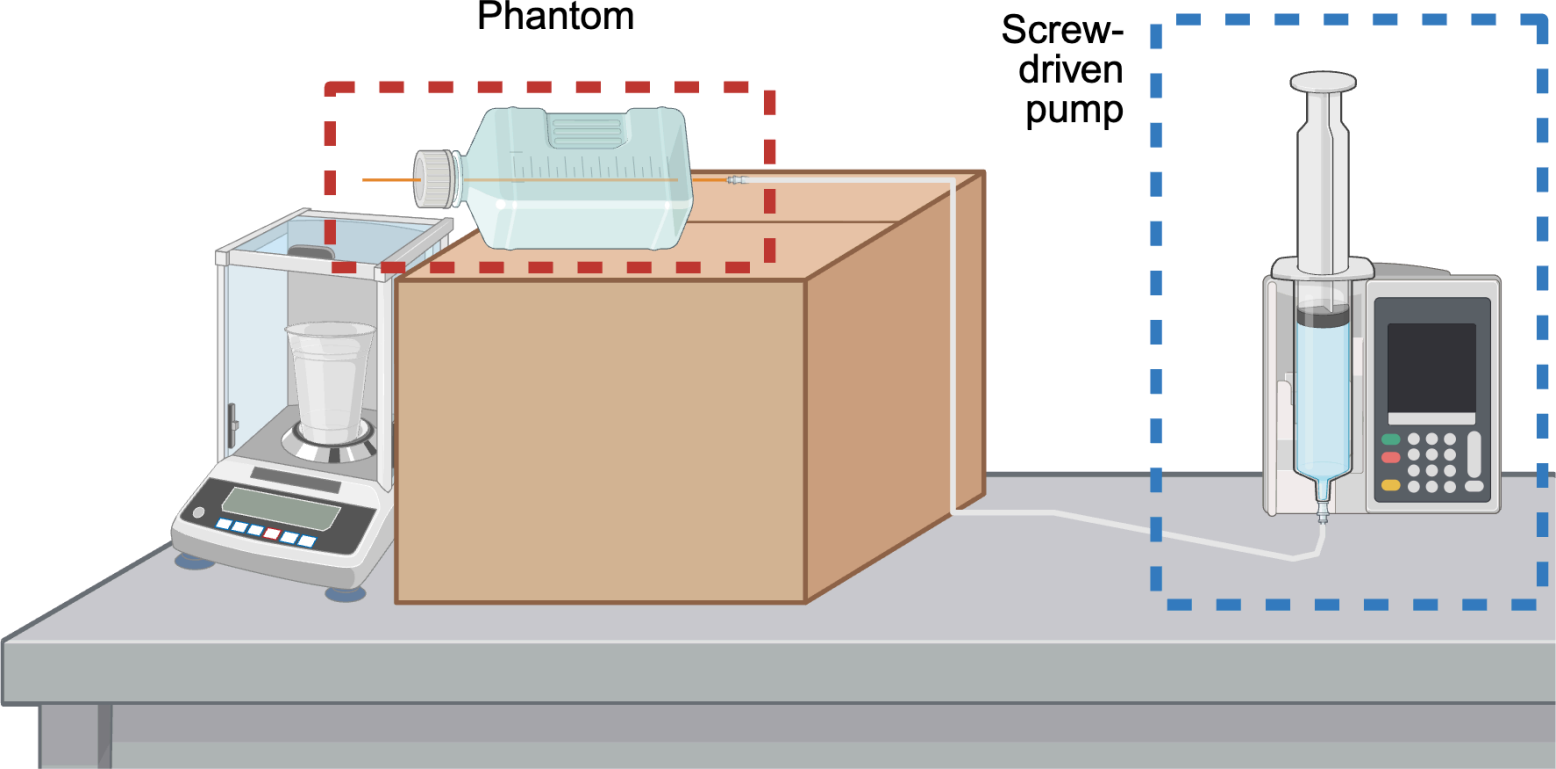
A schematic of the setup for calibration of the pump mechanism and single-tube phantom on an analytical balance.

We calibrated the combination of flow phantom and pump before data collection to ensure accurate and precise volumetric fluid delivery during subsequent testing (Figure 1). An analytical balance (AG204 DeltaRange; Mettler Toledo) was used to collect fluid output from the shunt system at 10 predetermined rates spanning the expected physiological range of CSF flow (0-22 mL per hour). The catheter was oriented flat on the benchtop to allow the liquid to drip freely, avoiding potential siphoning effects from gravitational pressure differences. Each pump setting was tested 3 times against the corresponding volumetric fluid output to evaluate the reproducibility of the pump’s flow.

### Protocol for PC-MRI Image Acquisition

PC-MRI flow acquisition is suggested with the following base sequence parameters: field of view of 30 × 30 mm, slice thickness of 10 mm, 2 signal averages, matrix size of 92 × 91, acquisition voxel dimensions of 0.20 × 0.20 mm^2^, reconstructed voxel dimensions of 0.10 × 0.10 mm^2^, compressed sensitivity encoding factor of 2, and velocity encoding of 1 cm per second [21].

**Figure 2.**
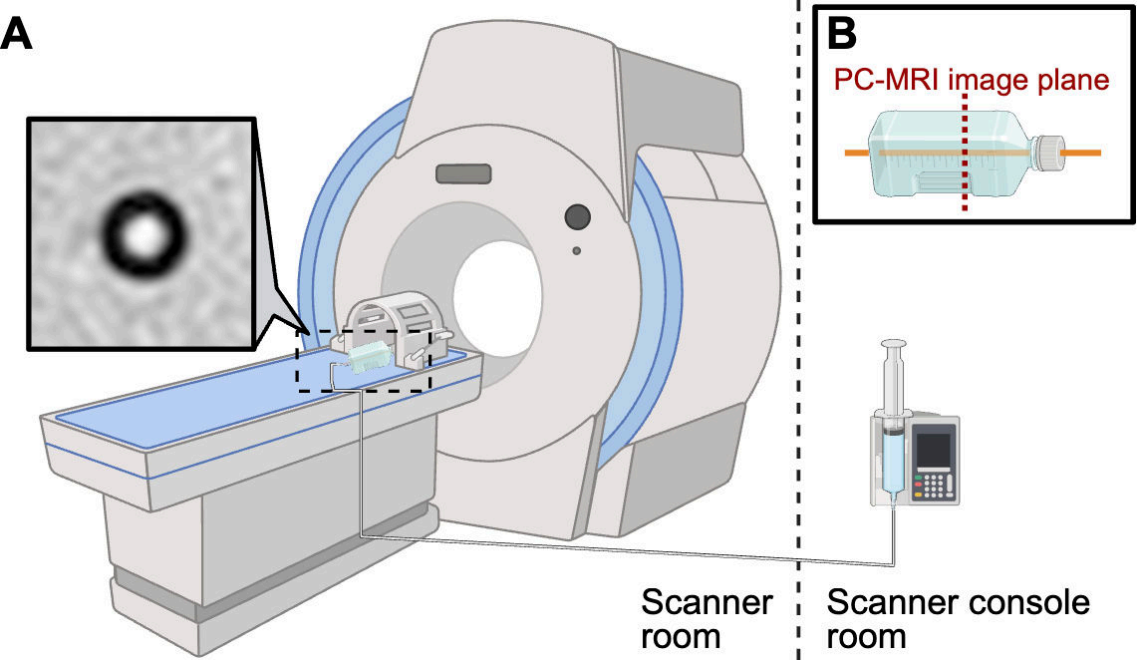
Scanner setup for phantom measurement: (A) schematic and magnitude image of the catheter lumen obtained on the single-tube phantom and (B) image plane through the phantom. PC-MRI: phase-contrast magnetic resonance imaging.

During image acquisition, the phantom is advised to be positioned parallel to the length of scanner bore and level with the MR bed. The screw-driven pump, located outside the MR room, is attached to the phantom, and connection tubing is carefully positioned to remain free of kinks or air (Figure 2). Triplanar (coronal, axial, and sagittal) T2-weighted images are used for PC-MRI slice localization, and PC-MRI image planes are placed orthogonally (transverse) to the direction of catheter flow such that the catheter lumen appears circular on phase-contrast images. The screw-driven pump is used to establish flow through the phantom at 10 benchtop-tested rates (0, 1, 3, 5, 8, 11, 14, 17, 19, and 22 mL/h) chosen for their overlap with the typical physiological range of CSF production. Four repeat PC-MRI images are obtained at each flow rate. Images are saved as Digital Imaging and Communications in Medicine files and exported to an external processing platform for flow measurement in a typical fashion.

To facilitate multi-institutional implementation, this acquisition and processing protocol is designed to be reproducible across centers with varying MR hardware configurations. Home institution plans for multiscanner calibration include repeating this procedure on 8 MR scanners in which field strength, gradient slew rate, gradient strength, radiofrequency coil type, model, and manufacturer vary (Table 1). Sites participating in multicenter data collection may apply this protocol to calibrate scanners for use in PC-MRI data acquisition.

**Table 1.** Variables that may affect phase-contrast magnetic resonance imaging (PC-MRI) measured flow rate.

Variable	Example parameters
Biological variation	
Flow	0-22 mL/h
Catheter type	Barium impregnated (Codman model [Integra LifeSciences] and Ares model [Medtronic]) Antibiotic-impregnated (Codman model and Ares model)
Image quality^a^	
Vendor or model	Siemens and Phillips
Field strength	0.55-7 T
RF^b^ coil type	8, 16, and 32 channels
Gradient slew rate	Covaries with field strength
Gradient strength	20-200 mT/m
TR^c^ and TE^d^	TR: 38-135 ms TE: 25-50 ms
Echo train length	0-3
Echo numbers	1
Slice thickness	5-10 mm
Number of averages	2-8
Acquisition matrix	Optimized for each manufacturer and system
Acquisition voxel dimensions	Optimized for each manufacturer and system
Bandwidth	24-230 Hz
Flip angle	5-45°
Acceleration factor	CSENSE^e^ or GRAPPA^f^
Scan duration	48-180 s
Acquisition^a^	
Angle	0-25°
VENC^g^	–1 to 1 cm/s
Shim location	Default shim One-quarter FOV^h^ shim offset Three-quarters FOV shim offset
Image processing	
ROI^i^ size	<1.3-mm inner diameter 1.3-mm inner diameter >1.3-mm inner diameter
ROI location	6 individuals will independently prescribe ROIs
Analytical method	Mean value Maximum value Parabolic model

a Image quality and acquisition parameters that can affect contrast-to-noise ratio on the PC-MRI image.

b RF: radiofrequency.

c TR: repetition time.

d TE: echo time.

e CSENSE: compressed sensitivity encoding.

f GRAPPA: generalized autocalibrating partially parallel acquisitions.

g VENC: velocity encoding gradient.

h FOV: field of view.

i ROI: region of interest.

### Analytic Methods of Flow Calculation

**Figure 3.**
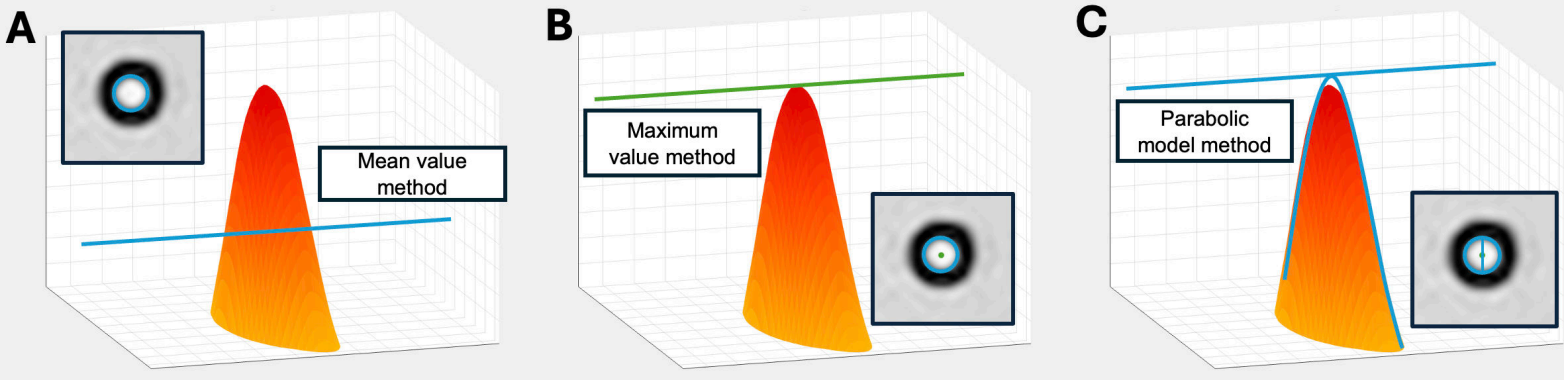
Methods for flow estimation: (A) mean value within a circular region of interest, (B) maximum value at the center of a parabolic flow profile, and (C) full parabolic fit of a laminar flow profile within the lumen.

We established three analytical approaches to measure volumetric flow from each PC-MRI image: (1) mean intensity within a circular region of interest prescribed over the catheter lumen; (2) maximum intensity within a small circular region of interest at the center of the catheter lumen, thereafter converted via a formula to total flow; and (3) full parabolic model of a laminar flow profile within the lumen (Figure 3). A description of each method of analysis and equations for flow calculation are provided in Multimedia Appendix 2.

### Regression Modeling and Statistical Comparison

For each scanner and hardware combination, linear regression will be used to evaluate the relationship between PC-MRI–derived flow rates and true flow outputs from the pump. An ANOVA will compare mean error between datasets, with a significance level set at α=.05. These statistical approaches will be repeated to evaluate the precision and accuracy of each analytical technique for flow measurement. Descriptive statistics will also be used to examine the effect of variables such as gradient strength, field strength, and slew rate on calibration measurements as these variables often covary with MR scanner.

### Multisite Data Collection: Design and Setting

We aim to validate the feasibility of implementing multisite PC-MRI data collection platforms by evaluating PC-MRI image quality and logistical factors such as scheduling feasibility, personnel training requirements, and processes for data transfer. Image quality will be reported as a proportion of analyzable scans within the dataset. Logistical factors surrounding barriers to personnel training or PC-MRI integration into scan protocols will be described individually by each site.

Patients who undergo a PC-MRI flow study will be included in the cohort regardless of age, etiology upon presentation, clinical setting (emergency department, inpatient, or outpatient), or MR hardware used during image acquisition. Patients with distal slit valves, valveless shunts, cystoperitoneal shunts, subdural shunts, and shunts with non–MR-resistant valves will be excluded. To maintain independence among data points, only the first PC-MRI flow measurement for each patient will be included in the analysis of shunt flow over a population of patients. Individuals with repeat PC-MRI measurements during unrelated clinical encounters will be analyzed separately to evaluate longitudinal trends in CSF flow.

### Data Collection and Management Plan

Clinical PC-MRI images will be collected across sites following standardized acquisition and processing protocols. A single PC-MRI sequence will be incorporated into routine and emergent full-length structural brain MRI exams for shunted patients at participating institutions. At each site, 2 independent data abstractors will manually review patient charts and clinical records according to predetermined standard operating procedures. Demographic, clinical, and imaging data (Multimedia Appendix 3) will be extracted from the electronic health records and collected using the REDCap (Research Electronic Data Capture; Vanderbilt University) tool [24]. PC-MRI images in which flow data cannot be measured (eg, low resolution, improper shim, and nonorthogonal image plane prescription) will be reported as “missing” and accompanied by documentation of image quality issues. Each institution will use local REDCap collection forms, and periodic queries for data validation will take place at 4-month intervals. For analysis, data will be merged between institutions via secure file transfer with Health Insurance Portability and Accountability Act (HIPAA) compliance. Datasets will be stored for 10 years after the study.

### Ethical Considerations

Home institution plans for PC-MRI imaging and data collection have been approved by the Children’s Hospital Los Angeles Institutional Review Board (IRB; CHLA-20-00041), and external sites will rely on local IRB approval along with data sharing agreements. As data for this study will be acquired retrospectively, a waiver of consent will be obtained; patient recruitment and informed consent before imaging will not be necessary. Identifying information will be correlated with unique study identification numbers stored on a local encrypted server.

### Safety Considerations

MRI is generally considered a safe imaging technique, with primary concerns related to implantable devices and projectile risks from ferromagnetic objects [25,26]. All patients will undergo standard MRI safety screening before imaging.

### Sample Size Estimates

To evaluate the feasibility of implementing a multisite PC-MRI data collection platform, we plan to enroll 100 participants across 3 sites. This target is based on our preliminary work and is expected to be feasible based on site selection and the number of eligible patients over the study period. A formal sample size calculation was not performed as the planned multisite study is focused on feasibility rather than hypothesis testing or estimation of effect sizes. The sample size is intended to ensure adequate experience with clinical data collection and will allow us to identify challenges related to PC-MRI acquisition, image quality, and flow measurement.

## Results

The shunt flow phantom was constructed in November 2024. Single-site phantom calibration began in April 2025 and concluded in December 2025. Registration of clinical data collection sites will take place between November 2025 and July 2026. Home institution IRB approval was obtained in January 2023 (before scanner calibration), and single-site data collection is ongoing. Multi-institutional clinical data collection will begin in July 2026 and continue until January 2027. Results and statistical analyses are expected by April 2027.

**Figure 4.**
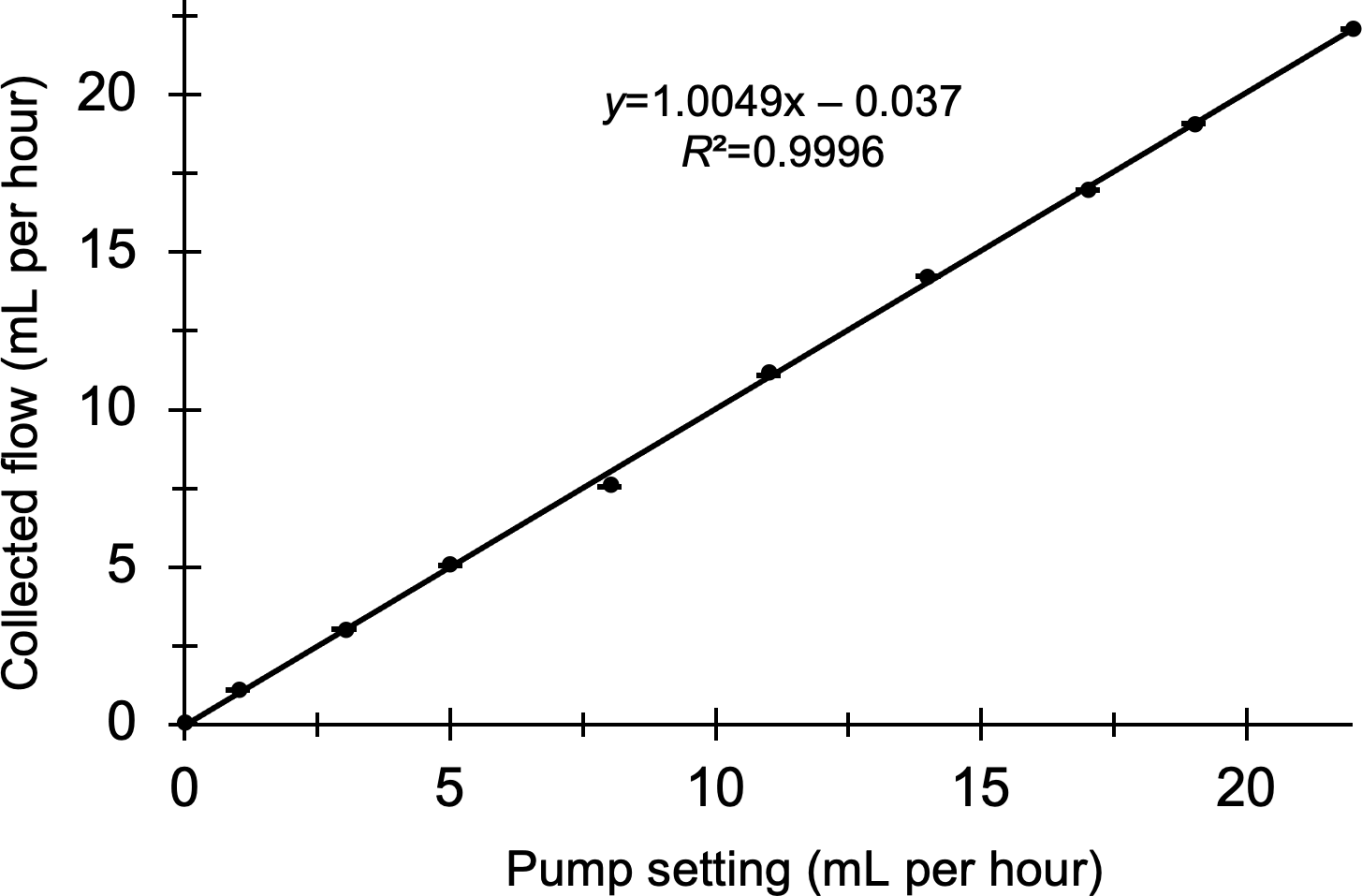
Syringe pump output setting (mL per hour; x-axis) vs true flow rate (mL per hour) calibrated on the analytical balance (y-axis).

Figure 4 shows the results of hardware calibration of the screw-driven syringe pump across appropriate flow values. Confirmation of pump accuracy is important before use in the MR scanner environment to establish correct baseline flow values and account for potential temporal drift in real pump output. The coefficient of determination (*R*^2^) between true and expected flow output from the pump in our case was 0.9997. Because the slope of the regression was close to 1 and the intercept was near 0, no calibration correction was applied.

## Discussion

### Standardizing PC-MRI With Reproducible Phantom Measurements

PC-MRI can provide additional utility in the diagnosis of shunt malfunction by offering a rapid and noninvasive approach to accurately quantify shunt flow. Our protocol presents a framework to calibrate individual MR scanners for accurate PC-MRI flow measurement in multi-institutional clinical data collection. Standardizing PC-MRI measurements and accounting for error introduced by variable MR hardware will allow individual institutions to perform appropriate calibration corrections. The ability to acquire reliable flow measurements between imaging centers can improve the validity of comparison between patient population flow values even when imaging is performed at different sites. Characterizing shunt flow across a large sample during multicenter data collection may improve the clinical assessment of shunt failure and our understanding of shunted hydrocephalus.

We designed a transportable and easy-to-use phantom that ensures magnetic homogeneity for reliable data acquisition. The single-catheter phantom and screw-driven syringe pump combination enables simple, consistent data collection across a physiological range of flows. To represent human shunt flow conditions, our phantom included a common catheter model used in patients at our institution, and water acted as a surrogate for CSF because both fluids have similar magnetic susceptibility and viscosity [27-29]. While CSF flow is known to vary with cardiac and respiratory cycles [30,31], PC-MRI measurements in this study will not be physiologically gated [21]. The potential influence of cardiac [32] or respiratory gating [33] on shunt flow measurement has not yet been studied and remains a potential area of future investigation.

Understanding the reproducibility of PC-MRI flow measurement across MR scanners is useful both to understand the influence of hardware parameters on measured shunt flow and to implement an appropriate calibration correction as needed. MR scanners can vary in field strength, slew rate, gradient strength, number of radiofrequency channel coils, model, and manufacturer at different institutions. This method for phantom model calibration will allow institutions to confirm the reliability of their MR scanners for accurate PC-MRI flow measurement. The protocol outlined in this work aims to streamline the implementation of PC-MRI by preemptively calibrating the scan sequence on a range of scanners and identifying challenges to multi-institutional data collection.

### Potential Limitations

As MR scanner time is primarily reserved for clinical use at most institutions, obtaining access for scanner calibration via phantom data collection remains a potential barrier. As PC-MRI images must be exported and externally processed by trained study personnel, the study may be limited by staffing constraints and processing delays. Outside of error introduced by MR hardware, it is important to note that human physiology (eg, susceptibility of the surrounding tissue [34] or altered pulsatility [30,35,36]) and movement within the scanner may provide additional potential variability. While this protocol aims to isolate the hardware-introduced variability in flow measurement, future research should investigate real-world sources of variation in flow measurement.

### Conclusions

This work details a protocol for multi-institutional clinical PC-MRI data collection. We also describe the construction of an easy-to-use phantom model of shunt flow and an interinstitutional procedure for scanner calibration to enable standardized collection of PC-MRI flow measurements. The tested phantom and pump combination exhibits accurate and precise outputs at both low and high flow rates while minimizing magnetic field inhomogeneities. An appropriate next step in progress at our institution and forthcoming at others is to generate scanner-specific calibration curves by acquiring PC-MRI data using the single-catheter phantom design. Widespread scanner calibration and validation of clinical feasibility will enable institutions to reliably implement PC-MRI, providing a quantitative method to evaluate shunt function in a broader patient population.

## Supplementary material

10.2196/85918 Multimedia Appendix 1 Phantom fabrication and pump evaluation.

10.2196/85918 Multimedia Appendix 2 Description of methods for flow calculation.

10.2196/85918 Multimedia Appendix 3 List of variables for extraction.

## Abbreviations:

CSF: cerebrospinal fluid
HIPAA: Health Insurance Portability and Accountability Act
IRB: institutional review board
MR: magnetic resonance
MRI: magnetic resonance imaging
PC-MRI: phase-contrast magnetic resonance imaging
REDCap: Research Electronic Data Capture
