# Supplementary material for: Noninvasive Measurement of Cerebrospinal Fluid Flow in Shunted Hydrocephalus: Protocol for Scanner Calibration and Multisite Data Collection
Source: JMIR Res Protoc. 2026 Feb 20;15:e85918. doi: 10.2196/85918 (PMC12923099; doi:10.2196/85918)
Supplement: Multimedia Appendix 1 [file resprot-v15-e85918-s001.docx]

**Supplemental Materials S1 – Material for Phantom Fabrication**

We tested 3 phantom designs, the one we report in the main text, and 2 others that allowed for simultaneous flow and slice plane prescription through 4 points along the catheter. This table provides the key materials and tools to build all 3 variations.

| **Product Name** | **Manufacturer** | **Image** |
| --- | --- | --- |
| 500 mL Narrow Mouth Polycarbonate Square Bottle | Nalgene (Rochester, NY) | See single tube phantom design below |
| ARES 1.3 mm Antibiotic-Impregnated Peritoneal Catheter (1200 mm length) | Medtronic (Minneapolis, MN) | 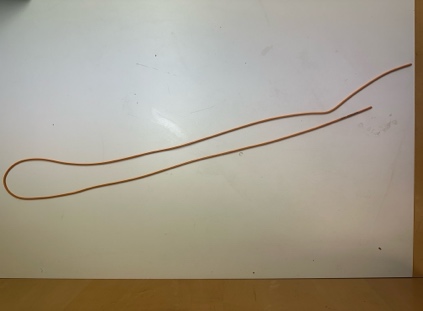 |
| Clear silicone waterproof sealant | Loctite (Rocky Hill, CT) | 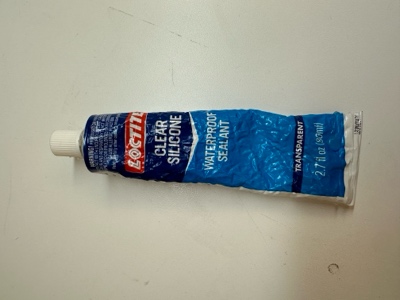 |
| Hand drill with 1/16-inch drill bit | Festool  (Wendlingen, Germany) | N/A |
| AG204 DeltaRange Analytical Balance | Mettler Toledo  (Columbus, OH) | 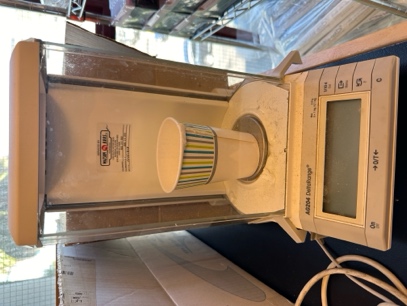 |
| Water (500 mL) | Tap | N/A |
| 360 in APPX 7.2 ml PUR smallbore Luer Lock tubing | ICU Medical Inc (San Clemente, CA) | 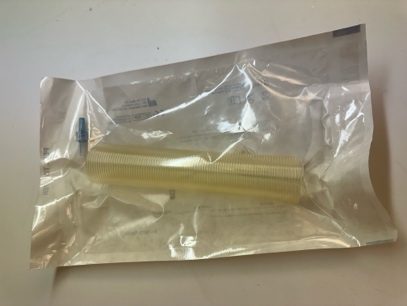 |
| 60 mL Luer Lock Syringe | Becton, Dickinson and Company (Franklin Lakes, CA) | 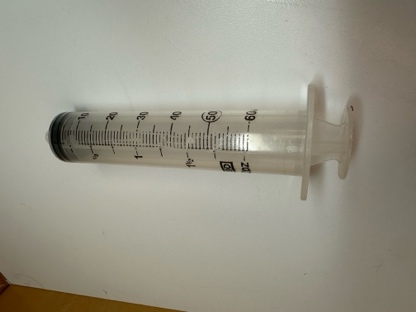 |
| Splitters  *Note: we evaluated but did not use these splitters in our final design. See “Bifurcated Fork Phantom Construction” in Phantom Fabrication, below.* | Value Plastics Inc (Fort Collins, CO) | 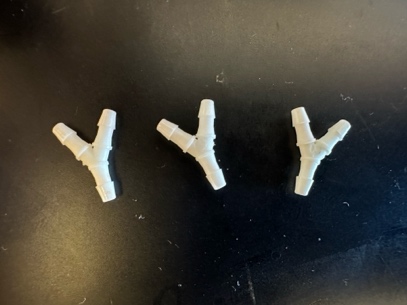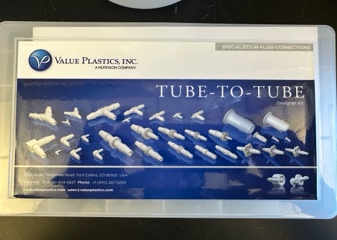 |
| Alaris PCU Syringe pump  (screw-driven infusion pump) | Becton, Dickinson and Company (Franklin Lakes, CA) | 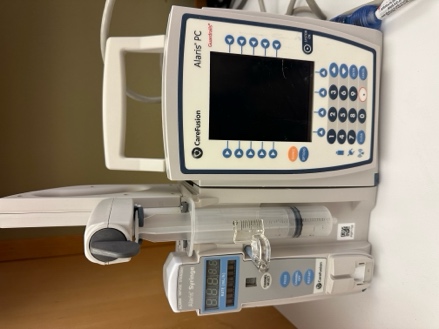 |
| Easy-load peristaltic pump  *Note: we evaluated but did not use this pump. See Pump Evaluation, below.* | MasterFlex (Gelsenkirchen, Germany) | 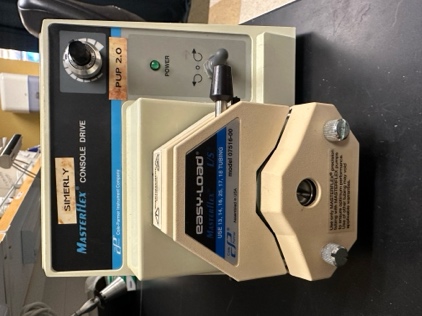 |
| 0.9% Sodium Chloride, 5,000 mL Arthrometric Bag *Note: we evaluated but did not use this as a gravity feed pump replacement. See Pump Evaluation, below.* | Baxter Healthcare Corporation (Deerfield, IL) | 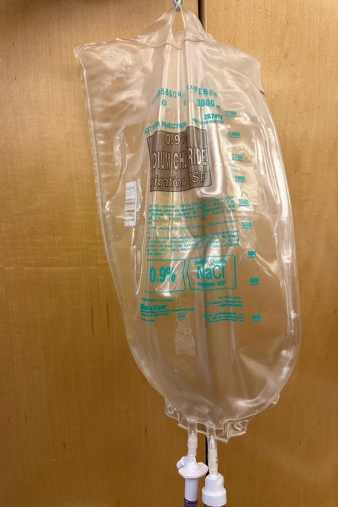 |

**Supplemental Materials S2 – Phantom Fabrication**

We fabricated several phantom types during our development process. These allowed us to explore different geometries which might facilitate our experiments. Ultimately, only one phantom design (single tube model, C) was selected. The 2 rejected phantom designs provided faster acquisition of additional repeat flow measurements but introduced additional error. Nevertheless, we provide these details to facilitate transparency and provide feedback on potentially suitable but rejected alternatives.

**
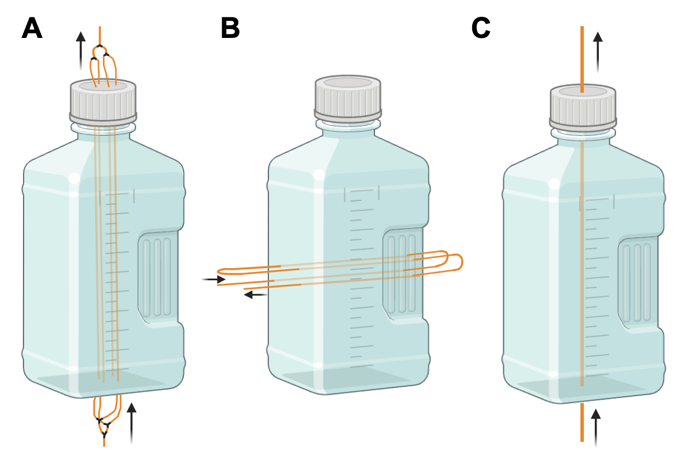
**

**Fig. S2** represents the 3 phantom models we evaluated. **A)** Bifurcated fork model, **B)** Double loop model, **C)** Single tube model

**Bifurcated Fork Phantom Construction (S2.A)**

This phantom was designed to enhance data collection which we attempted to enable with simultaneous measurement through four parallel shunt tubing segments. Ultimately this phantom was rejected because output flow was not equivalent between each of the 4 segments. This phantom is also prone to leaking or damage at the fragile bifurcations. Because of these limitations, we did not acquire experimental MR data, and do not recommend its use for scanner calibration.

1. Cut the catheter tubing into 4 equal lengths of tubing.
2. Drill 4 holes (1/16^th^ inch diameter) through the bottom of the bottle, and 4 holes through the cap. Ensure all holes are lined up directly above one another after tightening the cap.
3. Follow steps 2-7 from the ‘Single Tube Phantom Construction’ procedure to lock each tube in place.
4. Use 3 splitters to connect the ends of the 4 catheter portions into a single inflow, then connect this inflow to the Luer lock tubing and pump.

**Double Loop Phantom Construction (S2.B)**This phantom was designed to enhance data collection which we attempted to enable with simultaneous measurement through on series of shunt tubing which was passed through the imaging plane 4 times. Ultimately this phantom was rejected because the benefit from increased rate of data collection was offset by small differences in catheter orthogonality on a single imaging slice, where slice angle could not be adjusted individually for each catheter to optimize orthogonality, thus contributing to systematic error in flow measurement (**S4**). Further, the use of 4 catheters placed each catheter further from the center from the image and shim plane (30 × 30 mm field of view; 20 × 20 cm shim plane), occasionally leading to poor shim quality at the site of the catheter.

1. Drill 4 holes (1/16^th^ inch diameter) through each side of the Nalgene bottle and ensure each pair of holes is directly across from the other.
2. Weave the catheter through the aligned holes.
3. Seal catheter in place at all openings and then fill the bottle with water through the top.

**Single Tube Phantom Construction (S2.C)**

This phantom was designed to be the simplest to build and the most robust in terms of risk of mechanical damage and precision during data collection (**S4**). We recommend this phantom.

1. Drill a hole (1/16^th^ inch diameter) through the center of the bottle’s base, and another through the center of the bottle cap. Ensure these holes are aligned vertically.
2. Feed the catheter through each of the holes so that it transverses the bottle longitudinally. Position the catheter so equal lengths extend from both the top and bottom of the bottle.
3. Ensure the catheter is straight and untwisted, then secure it in place on the bottom of the bottle only using silicone sealant.
4. Allow the sealant to dry for 45 minutes before proceeding.
5. Fill the bottle fully with water, taking care to avoid any air bubbles trapped inside, and screw on the bottle’s cap (with the catheter inserted through the hole).
6. Add more water to the hole at the top of the bottle to remove any remaining air bubbles.
7. Hold each end of the catheter to ensure the tubing is free from kinks and taut but not stretched or bent. Secure the catheter at the top-hole using silicone sealant. Maintain this position for 45 minutes while the sealant dries. Releasing the catheter too early may cause the tubing within the bottle to bend and come loose from the sealant.

**Supplemental Materials S3 – Pump Evaluation**

We compared the use of a screw-driven infusion pump, peristaltic pump, and IV bag to adjust the rate of fluid flow through our phantom system. Each pump was attached to one end of the shunt catheter using the small-bore Luer lock tubing. Flow settings from each pump were validated on the benchtop by measuring flow output on an analytical balance. Calibration revealed that the screw driven infusion pump achieved both precision and accuracy across all flow rates tested, whereas the peristaltic pump and IV bag struggled to maintain precision at lower flow settings. Based on these findings, we recommend using a screw driven infusion pump for all phantom calibrations.

**Supplemental Materials S4 – Phantom Imaging**

**S4 Figure:**

**
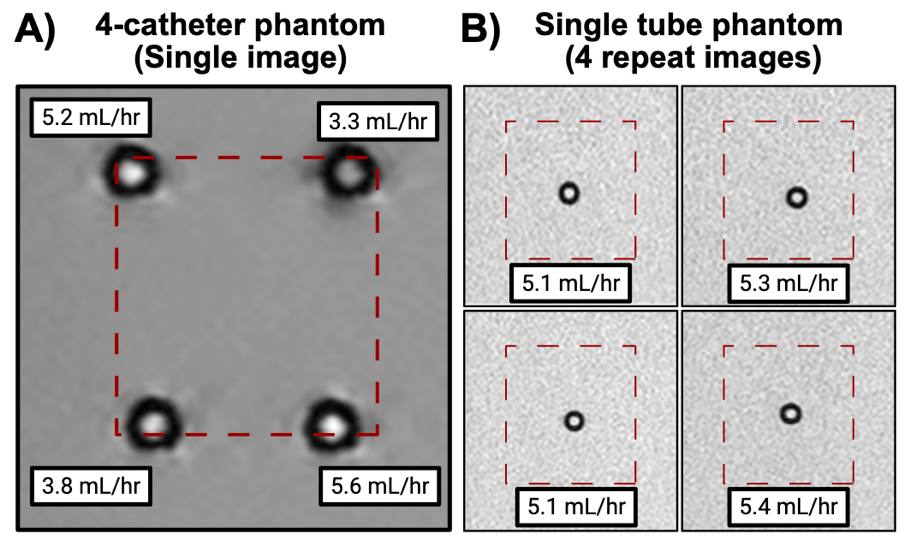
**

**Fig. S4** represents the magnitude image and overlayed location of the shim field (red dotted line) across two phantom models. The PC-MRI measured flow rates from each catheter are also provided.
