# Supplementary material for: Noninvasive Measurement of Cerebrospinal Fluid Flow in Shunted Hydrocephalus: Protocol for Scanner Calibration and Multisite Data Collection
Source: JMIR Res Protoc. 2026 Feb 20;15:e85918. doi: 10.2196/85918 (PMC12923099; doi:10.2196/85918)
Supplement: Multimedia Appendix 2 [file resprot-v15-e85918-s002.docx]

**Description of Methods for Flow Calculation**

The mean intensity method approximates volumetric flow using the average intensity within the established ROI. A circular ROI (1.3 mm diameter) is centered in the catheter lumen on the magnitude image (structural visualization is optimized on magnitude image), with subsequent ROI transfer to the phase image in the same coordinate reference frame (copy and paste function in Horos [Purview, Annapolis, MD]). Intensity measurements are converted into velocity values as in Equation 1:

**(Eq. 1)** $v =\frac{V_{enc, max} - V_{enc, min}}{I_{max} - I_{min}}\left( I_{mean} - I_{max} \right) + V_{enc, max}$

where $v$ is the velocity, *V_enc, max_* is the maximum velocity encoding gradient used (1), *V_enc, min_* is the minimum velocity encoding gradient (-1), *I_max_* is the maximum voxel intensity on the entire phase image, *I_min_* is the minimum voxel intensity on the phase image, and *I_mean_* is the mean intensity within the ROI. Velocity measurements are converted into volumetric flow rates (in cc/hr) using dimensional analysis (Equation 2):

**(Eq. 2)** $Q= v_{mean}\cdot A$

where $Q$ is the volumetric flow rate, $v_{mean}$ is the fluid velocity, and *A* is the cross-sectional area of the ROI.

As shunt flow has been found to retain a laminar flow profile [1], the maximum velocity ${(v}_{max})$ of laminar fluid flow can be used to approximate volumetric flow rate over the entire lumen by Equation 3:

**(Eq. 3)** $Q= \frac{v_{max} \cdot A}{2}$

The maximum velocity measurement is obtained directly by calculating the highest intensity voxel within the lumen ROI (using MATLAB [MathWorks, Natick, MA]) and can also be estimated more robustly with a parabolic fit to the full laminar flow profile within the lumen. The parabola is generated by sampling intensity values along a lumenal diameter cross-section, passing through the center point of the lumen and modeled using MATLAB’s *polyfit* function from the Curve Fitting Toolbox [2].

**References**

1. Ha JH, Borzage MT, Vanstrum EB, Doyle EK, Upreti M, Tamrazi B, et al. Quantitative noninvasive measurement of cerebrospinal fluid flow in shunted hydrocephalus. J Neurosurg. 2024;140(4):1117-28.

2. The MathWorks I. Curve Fitting Toolbox User's Guide. Natick, Massachusetts: The MathWorks, Inc.; 2024.
