# Supplementary material for: Noninvasive Measurement of Cerebrospinal Fluid Flow in Shunted Hydrocephalus: Protocol for Scanner Calibration and Multisite Data Collection
Source: JMIR Res Protoc. 2026 Feb 20;15:e85918. doi: 10.2196/85918 (PMC12923099; doi:10.2196/85918)
Supplement: Multimedia Appendix 3 [file resprot-v15-e85918-s003.docx]

**Supplemental Table – List of Variables for Extraction**

| **Category** | **Individual Variables to be Extracted** | |
| --- | --- | --- |
| Demographic Data | Age at clinical encounter^a^ | |
|  | Prematurity? (yes/no) | |
|  | Sex (Male/Female) | |
|  | Race/Ethnicity | |
|  |  | Hispanic/Latino |
|  |  | Non-Hispanic – White |
|  |  | Non-Hispanic - Black |
|  |  | Other |
| Historical Data | Etiology/Etiology code | |
|  |  | Hemorrhage |
|  |  | Meningitis |
|  |  | Arachnoid cyst |
|  |  | Choroid plexus papilloma |
|  |  | Hydrocephalus |
|  |  | Chiarai malformation |
|  |  | Other |
|  | Date of initial shunt placement | |
|  | Shunt Malfunction (yes/no) | |
|  | Shunt Catheter Inner Diameter | |
|  | Shunt Type | |
|  |  | Codman Antibiotic Impregnated |
|  |  | Ares Antibiotic Impregnated |
|  |  | Codman Barium Impregnated |
|  |  | Ares Barium Impregnated |
|  | Valve type and setting | |
|  |  | Sophiya (P/NP) |
|  |  | Strata (P/NP) |
|  |  | Deltya (P/NP) |
|  |  | Certas (P/NP) |
|  |  | Codman (P) |
|  | Number of shunt revisions | |
| Clinical Data | Height^a^ | |
|  | Weight^a^ | |
|  | Head Size^a^ | |
|  | Atrium Size^a^ | |
|  | Length of stay^a^ | |
|  | Symptoms upon presentation | |
|  |  | Headache |
|  |  | Nasuea/Vomiting |
|  |  | Fever |
|  |  | Irritability |
|  |  | Sleepiness |
|  |  | Loss of Balance |
|  |  | Constipation |
|  |  | Bradycardia |
|  |  | Swollen shunt tract |
|  |  | Change in heart size |
|  |  | Papilledema |
|  |  | Full Fontanel |
|  |  | Downward Gaze |
|  |  | Decline in Exam |
|  |  | Ventricular Size change |
|  | Cardiovascular conditions? (y/n) | |
|  |  | Congenital heart defects |
|  |  | Coronary heart disease |
|  |  | Heart Failure |
|  |  | Arrhythmias |
|  |  | Cardiomyopathy |
|  |  | Hypertension |
|  |  | Prior cardiac surgery or intervention |
|  |  | Other cardiovascular condition |
| Imaging data | Time and Date of scan | |
|  | Scan location (Inpatient/Outpatient) | |
|  | MR Scanner Information | |
|  |  | Make/model |
|  |  | Field strength |
|  |  | Gradient strength/gradient slew rate |
|  |  | Acceleration factor |
|  | Rating of image quality | |
|  | PC-MRI measured flow rate^a^ | |
|  | Frontal Horn diameter^a^ | |
|  | Ventricular size change? | |
|  |  | No Change |
|  |  | Decreased |
|  |  | Small increase |
|  |  | Large increase |
| Clinical findings and procedural results | Shunt tap result | |
|  |  | N/A |
|  |  | No flow returned |
|  |  | Hesitant flow returned |
|  |  | Consistent Flow returned |
|  |  | Elevated ICP |
|  |  | Normal ICP |
|  | Nuclear med performed? (yes/no) | |
|  |  | Result? (confirmed flow/no flow) |
|  | Discharge diagnosis code | |
|  | Confirmed shunt malfunction? (yes/no) | |

^a^Denotes variable which will be measured continuously
